# Comment on Rosa et al. Optic Nerve Drusen Evaluation: A Comparison between Ultrasound and OCT. *J. Clin. Med.* 2022, 11, 3715

**DOI:** 10.3390/jcm12175607

**Published:** 2023-08-28

**Authors:** Kurt J. Schimmelbusch, Frederick T. Collison

**Affiliations:** 1 Chicago College of Osteopathic Medicine, Midwestern University, Downers Grove, IL 60515, USA; 2 Chicago College of Optometry, Midwestern University, Downers Grove, IL 60515, USA

We read with interest the paper by Rosa et al., entitled “Optic Nerve Drusen Evaluation: A Comparison between Ultrasound and OCT”, published in June of 2022 [1]. We agree with the authors on the importance of distinguishing optic disc drusen (ODD) from optic disc edema. However, we draw attention to Figures 3 and 4, which do not seem to represent the enhanced depth imaging (EDI) capacity of optical coherence tomography (OCT).

While the Methods of this article state that the enhanced-depth mode was used for the cross-sectional imaging of the optic discs, this claim is contradicted by the appearance of both OCT figures in the paper, neither of which bears the features of enhanced-depth imaging, such as a visible choroid-scleral junction. This might explain the group's finding of 51% sensitivity for EDI-OCT—a number which is more similar to the pre-EDI-era accuracy of OCT relative to ultrasound [2].

The authors also limit the diagnosis of ODD to ultrasound findings in their definition, which assumes a 100% accuracy for this modality and does not establish a basis for an objective comparison with OCT in statistical analysis [3]. The authors refer to b-scan ultrasound as the “gold-standard” for ODD diagnosis, which differs from the most recent recommendation of the ODDS consortium [4].

We note a further departure from the ODDS consortium in the Results, wherein peripapillary hyper-reflective ovoid mass-like structures (PHOMS) are described as markers for ODD. PHOMS can be found in several conditions (including the main differential, papilledema), most of which have no relationship with optic disc drusen [5].

We recommend further study, including but not limited to the utilization of the enhanced depth features of EDI-OCT, before conclusions are drawn on the ability of b-scan ultrasound to outperform OCT.
